# Relationship between postoperative lordosis distribution index and adjacent segment disease following L4-S1 posterior lumbar interbody fusion

**DOI:** 10.1186/s13018-020-01630-9

**Published:** 2020-04-03

**Authors:** Guoquan Zheng, Chunguo Wang, Tianhao Wang, Wenhao Hu, Quanbo Ji, Fanqi Hu, Jianrui Li, Surendra K. Chaudhary, Kai Song, Diyu Song, Zhifa Zhang, Yongyu Hao, Yao Wang, Jing Li, Qingyuan Zheng, Xuesong Zhang, Yan Wang

**Affiliations:** 1 Department of Orthopedics, Chinese PLA General Hospital, Fuxing Road, Beijing, China; 2 Tongji University School of Medicine, Shanghai, China

**Keywords:** Adjacent segment disease, Posterior lumbar interbody fusion, Lordosis distribution index, Lower lumbar lordosis, Lumbar lordosis

## Abstract

**Background:**

Adjacent segment disease (ASD**)** is an acknowledged problem of posterior lumbar interbody fusion (PLIF). Many studies have been reported concerning the role of lordosis distribution index (LDI) in spinal biomechanics. However, few reports have been published about the impact of LDI on ASD following L4-S1 PLIF.

**Methods:**

The study enrolled 200 subjects who underwent L4-S1 PLIF for degenerative spine disease from 2009 to 2014. The average follow-up term was 84 months. Several lower lumbar parameters were measured, including lower lumbar lordosis (LLL), lumbar lordosis (LL), pelvic incidence (PI), and LDI on the pre and postoperative radiograph. Perioperative information, comorbidities, and operative data were documented. Kaplan-Meier curves were plotted for the comparisons of ASD-free survival of 3 different types of postoperative LDI subgroups.

**Results:**

The incidence of ASD was found to be 8.5%. LL and LLL increased by 3.96° (38.71° vs 42.67°; *P* < 0.001) and 3.60° (26.22° vs 28.82°; *P* < 0.001) after lower lumbar fusion surgery, respectively. Lordosis distribution index (LDI) increased by 0.03 (0.66 vs 0.69, *P* = 0.004) postoperatively. A significant difference (*P* = 0.001) was observed when comparing the incidence of ASD among postoperative LDI subgroups. The Kaplan-Meier curves showed a marked difference in ASD-free survival between low and moderate LDI subgroup (log-rank test, *P* = 0.0012) and high and moderate LDI subgroup (log-rank test, *P* = 0.0005).

**Conclusion:**

Patients with abnormal postoperative LDI were statistically more likely to develop ASD than those who had normal postoperative LDI. Moreover, patients with low postoperative LDI were at greater risk for developing ASD than those with high postoperative LDI over time.

## Introduction

Posterior lumbar interbody fusion (PLIF) with pedicle screw fixation has been widely applied to treat various lumbar spinal disorders including lumbar spinal stenosis, lumbar disc herniation, degenerative spinal deformity, instability, spondylolisthesis, and vertebral compression fracture. Despite the capability of PLIF with pedicle screw fixation to provide solid instrumentation and to achieve satisfactory clinical effects, it has a potential to alter the normal biomechanics of the spine and to accelerate the degenerative process of adjacent unfused segments, thus increasing the risk of developing adjacent segment disease (ASD) [1–6]. ASD is one of the widely acknowledged problems of PLIF which could cause various complications, for example, herniated nucleus pulposus, spondylolisthesis, stenosis, hypertrophic facet arthritis, and scoliosis [5, 7, 8].

At present, there have been plenty of studies suggesting the risk factors for the development of ASD after lumbar spinal fusion, including age, gender, obesity, body mass index (BMI), fusion length, osteoporosis, laminectomy performed adjacent to a segment, preoperative segmental instability at the adjacent level, preexisting degenerated disc prior to fusion, and excessive disc height distraction [9–12]. In addition, lower lumbar interbody fusion surgeries represent the major part of the spinal surgeries in the clinical practice of a spine surgeon [13]. The total lumbar lordosis consists primarily of the upper-arc lordosis of L1–L3 and lower-arc lordosis of L4-S1. Lower lumbar lordosis (LLL), which is defined as the angle between the superior end plate of L4 and S1, accounts for two-thirds of the total lumbar lordosis [14, 15]. Lordosis distribution index (LDI), which is indicated as L4-S1 lordosis/L1–S1 lordosis × 100%, determines the magnitude of lower-arc lordosis relative to the total lordosis [16]. Up to now, there remain few studies demonstrating the impact of LDI on ASD following lower lumbar spine surgery. Therefore, it is essential to identify the factors in reducing the incidence of ASD in patients with lower lumbar spine.

This study is purposed to investigate the association between postoperative LDI and ASD following L4-S1 posterior lumbar interbody fusion with pedicle screw fixation for spinal degenerative diseases, and provide practical guidance on surgical planning for a spine surgeon to improve clinical outcomes while further lowering medical costs.

## Materials and methods

### Subjects and surgical procedure

This is a retrospective study, involving a total of 215 consecutive patients who received treatment for spinal degenerative pathologies at the Chinese PLA General Hospital during a 5-year period from 2009 to 2014. The protocol was granted approval from the Research Ethics Committee of the hospital. The patients were enrolled to meet the following inclusion criteria: degenerative disorders of lower lumbar spine such as lumbar disc herniation and symptomatic lumbar spinal stenosis with severe lower back pain and radiculopathy. All of the patients were treated with L4-S1 PLIF and bilateral lumbar laminectomy of vertebrae L4–5 using a PEEK cage inserted into intervertebral space. The cage was placed after the disc space was cleaned and the autograft bones crumbled from resected lamina were put into the space. The minimum follow-up period was 19 months with imaging collection including lumbar plain anteroposterior and lateral X-ray obtained prior to surgery, immediately following surgery. CT and MRI scans conducted before surgery, the instant a new onset of symptoms appeared and at the final follow-up visit. The study excluded patients who were diagnosed with spinal neoplasm, trauma, hip joint disease, infection, compression fracture of vertebra, inflammation, rheumatoid arthritis, and isthmic spondylolisthesis. The patients suffering scoliosis with a Cobb angle ≥ 10°, preexisting lumbar fusion, or laminectomy surgeries, preoperative spondylolisthesis ≥ 3 mm at the adjacent level, and severe adjacent intervertebral disc degeneration of Pfirrmann classification V were also excluded from this study [17]. Therefore, a total of 200 patients (89 men and 111 women) were involved in this study with an age of 64.8 ± 8.5 years on average (range, 36 years to 85 years). The average follow-up term was 84 months (*P*_25_~*P*_75_, 70~98 months).

ASD refers to the pathologic conditions as observed on radiograph with clinical symptoms that may require an additional surgical intervention treat neurological symptoms at the level adjacent to previous fusion [18]. Radiographic ASD is defined by segmental kyphosis more than 10°, the development of anterolisthesis or retrolisthesis of more than 3 mm and the deterioration in the Pfirrmann classification (grades I–V) or Imagama’s classification (grades I–IV) of one grade or greater progression at the level adjacent to a previous spinal fusion [19–21]. The clinical ASD is considered to be adjacent disc degeneration causing various newly developed symptoms, such as stenosis, instability, and neurological abnormality [22].

### Radiographic evaluation and clinical data collection

**Fig. 1:**
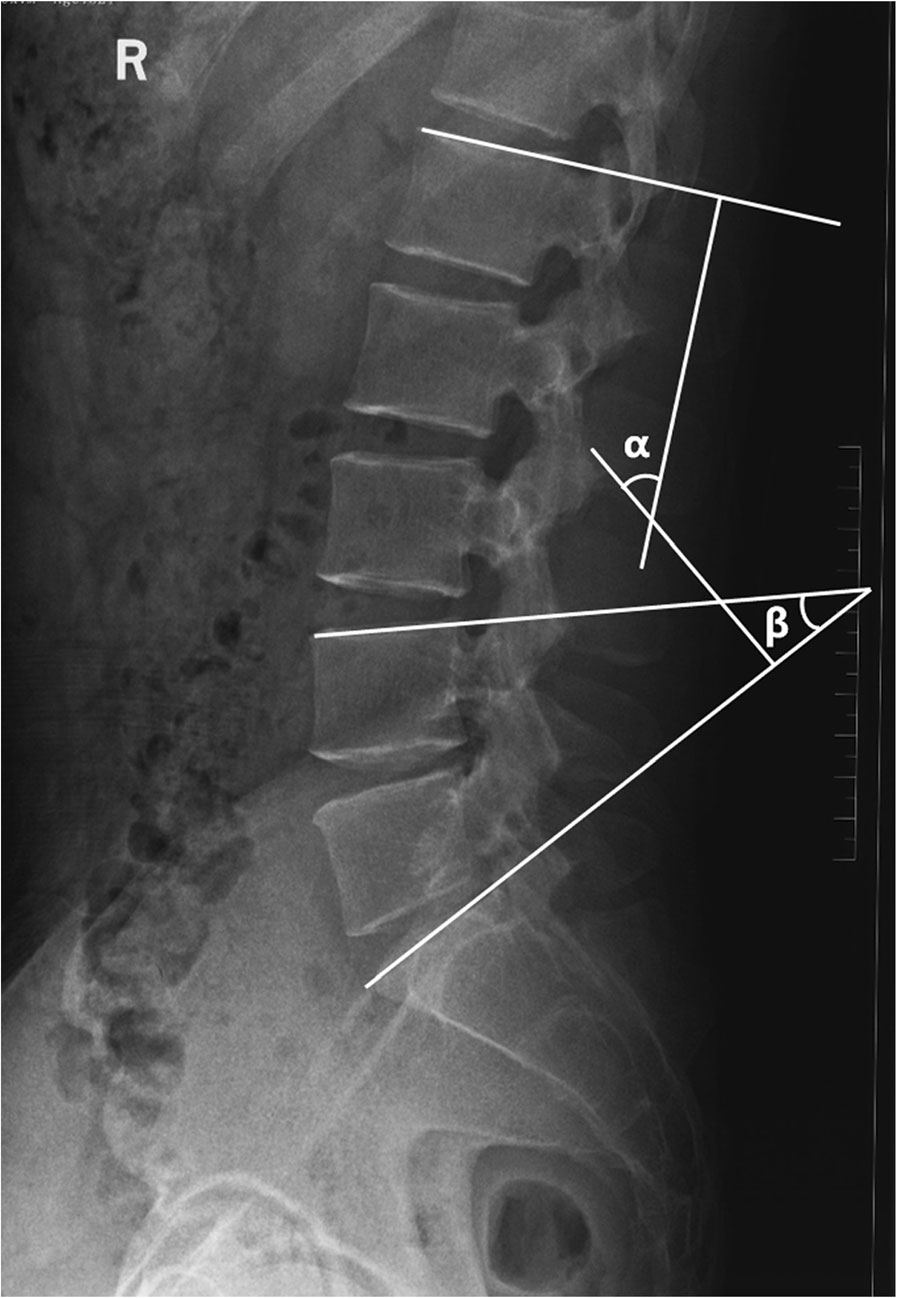
Measurement of lower lumbar spinal parameters. LL (α) is the angle between the superior endplate of L-1 and S-1. LLL (β) is the angle between the superior end plate of L4 and S1. LDI equals to β/α × 100%

Lumbar lordosis (LL) is determined by the angle between the superior end plate of L1 and S1 on the neutral lateral X-ray image. Lower lumbar lordosis (LLL) is measured from the superior end plate of L4 to the superior endplate of S1 on lumbar plain lateral X-ray using the Cobb method. Pelvic incidence (PI) is defined as the angle between the line perpendicular to the sacral plate at its midpoint and the line connecting this point to the femoral heads axis. Lordosis distribution index (LDI) indicated using the following formula: lower lumbar lordosis/lumbar lordosis × 100% (Fig. 1). Based on our current clinical experiences, each patient was positioned on the lateral decubitus position when a radiograph was taken pre and postoperatively. The patient was kept in a neutral position, with the trunk neither flexed nor stretched. All of the measurements were performed twice by a trained spine surgeon blinded to the clinical outcomes with the assistance of Surgimap (version 2.3). Then the mean values were recorded for subsequent analysis. LDI was split into 3 subgroups. An LDI less than 50% was treated as hypolordotic maldistribution (low LDI), 50–80% as aligned (moderate LDI) and over 80% as hyperlordotic maldistribution (high LDI) [16]. The perioperative information included demographic variables, comorbidities, and operative data.

### Statistical analysis

In accordance with Shapiro-Wilk normality tests, continuous variables are indicated as mean± standard deviation or *P*_50_ (*P*_25_~*P*_75_). SPSS (version 23) for windows was performed for subsequent statistical analysis. The Pearson *χ*^2^ test or Fisher’s exact test was carried out for the comparison of gender, BMI intergroup, comorbidities, pre and postoperative LDI subgroup between ASD and non-ASD group. The nonparametric test of Mann-Whitney *U* test was conducted for the follow-up period, intraoperative blood loss, operation time, and hospital stay. The independent-samples *t* test was carried out for radiographic parameters of the lumbar spine. The paired-samples *t* test was performed to compare changes in the lower lumbar parameters before and after operation. Kaplan-Meier curves were plotted and compared by log-rank tests. An α level of 0.05 was considered to be statistically significant.

## Results

### Subjects and surgical procedure

**Fig. 2:**
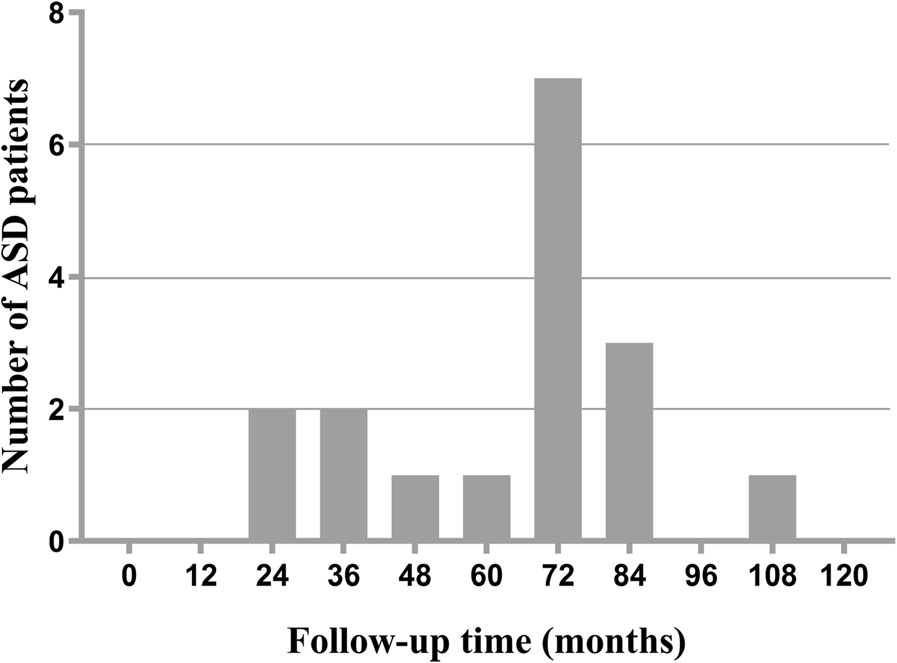
Frequency distribution of follow-up time of adjacent segment disease (ASD) patients after L4-S1 posterior lumbar interbody fusion

ASD after L4-S1 PLIF for the degenerative disease of lower lumbar spine was developed in 17 out of 200 patients (8.5%) during the final follow-up. The segment lesions were identified at the level above the fusion and all of them were assigned to the ASD group. In the ASD group, 7 male and 10 female patients were included, with an age of 66.1 ± 5.1 on average. The mean follow-up period was 62.8 ± 24.6 months. The average BMI at admission was 27.04 ± 3.00 kg/m^2^. In the non-ASD group, 82 male and 101 female patients were included, with an average age of 64.7 ± 8.7. The average follow-up duration was 85 months (72 months~99 months). The average BMI at admission was 23.87 ± 3.33 kg/m^2^. In 79 patients, BMI ≥ 25 kg/m^2^, of whom 13 patients (12.87%) ended up developing ASD. In 121 patients, BMI < 25 kg/m^2^, of whom 4 patients (3.31%) were finally diagnosed as having ASD. Table 1 presents the comparisons of basic characteristics of patients between ASD and non-ASD groups. Statistical analysis was conducted to identify the significant difference in BMI (23.87 vs 27.04, *P* < 0.001), BMI intergroup (23.5% vs 76.5%, *P* = 0.001) and the average follow-up period (85.0 vs 62.8, *P* < 0.001). Nevertheless, there was no statistically significant difference observed between the two groups in such basic variables as age, gender, comorbidities, hospital stay, intraoperative blood loss, and operation time. Figure 2 shows the frequency distribution of follow-up time for ASD patients after L4-S1 PLIF.

**Table 1:** Comparisons of basic characteristics of patients between ASD and non-ASD group

	Total (***n*** = 200)	Non-ASD	ASD	***P***
Age, years	64.8 ± 8.5	64.7 ± 8.7	66.1 ± 5.1	0.315
Sex, *n* (%)				0.773
Male	89 (44.5)	82 (44.8)	7 (41.2)	
Female	111 (55.5)	101 (55.2)	10 (58.8)	
BMI	24.14 ± 3.42	23.87 ± 3.33	27.04 ± 3.00	< **0.001**
BMI group, *n* (%)				**0.001**
BMI < 25 kg/m^2^	121 (60.5)	117 (63.9)	4 (23.5)	
BMI ≥ 25 kg/m^2^	79 (39.5)	66 (36.1)	13 (76.5)	
Comorbidities, *n* (%)				
Hypertension	131 (65.5)	119 (65.0)	12 (70.6)	0.645
Diabetes Mellitus	57 (28.5)	51 (27.9)	6 (35.3)	0.713
Coronary artery disease	97 (48.5)	90 (49.2)	7 (41.2)	0.528
COPD	39 (19.5)	34 (18.6)	5 (29.4)	0.448
Hospital stay (days)	9.0 (7.0~11.0)	9.0 (7.0~11.0)	8.0 (6.0~11.5)	0.757
Follow-up period (months)	84.0 (70.0~98.0)	85.0 (72.0~99.0)	62.8 ± 24.6	< **0.001**
Intraoperative blood loss (ml)	290 (220~380)	290 (220~380)	294.7 ± 111.0	0.920
Operation time (hours)	4.45 (3.70~5.30)	4.50 (3.70~5.30)	4.35 ± 0.87	0.525

Mann-Whitney *U* test, Pearson *χ*^2^ test, independent-samples *t* test

*ASD* adjacent segment disease, *BMI* body mass index, *COPD* chronic obstructive pulmonary disease

### Radiographic evaluation and clinical data collection

In the radiographic evaluation, the lower lumbar spinal parameters as measured using both pre and postoperative radiographs are shown in Table 2. LL and LLL increased by 3.96° (38.71° vs 42.67°, *P* < 0.001) and 3.60° (26.22° vs 28.82°, *P* < 0.001) following the lower lumbar fusion surgery, respectively. Moreover, LDI rose by 0.03 (0.66 vs 0.69, *P* = 0.004) postoperatively. In Table 3, the comparisons of spinopelvic parameters and LDI with and without ASD groups are demonstrated. There is no statistical significance found in radiographic parameters of pre LL (37.17° vs 38.85°, *P* = 0.341), pre LLL (23.71° vs 26.36°, *P* = 0.177), the value of pre LDI (0.65 vs 0.66, *P* = 0.682), post LL (40.35° vs 42.88°, *P* = 0.241), post LLL (27.69° vs 28.93°, *P* = 0.410), and the value of post LDI (0.71 vs 0.68, *P* = 0.578) and PI (55.49 ± 8.22° vs 53.26 ± 7.13°, *P* = 0.251). When the pre and postoperative LDI subgroups were compared between ASD and non-ASD groups, the number of patients with low LDI pre and postoperatively in the ASD group was 2 (11.8%) and 4 (23.5%), respectively. The number of patients with moderate LDI pre and postoperatively in the ASD group was 13 (76.4%) and 6 (35.3%), respectively. The number of patients with high LDI pre and postoperatively in the ASD group was 2 (11.8%) and 7 (41.2%), respectively. Consequently, a significant difference was observed in the postoperative LDI subgroups (*P* = 0.001) but not in the preoperative LDI subgroups (*P* = 0.252).

**Table 2:** Changes in pre and postoperative radiographic parameters

Radiographic parameters	Pre op	Post op	***P***
Lumbar lordosis (°)	38.71 ± 6.96	42.67 ± 8.51	< **0.001**
Lower lumbar lordosis (°)	25.22 ± 4.82	28.82 ± 5.90	< **0.001**
Lordosis distribution index	0.66 ± 0.10	0.69 ± 0.13	**0.004**

Paired-samples *t* test

**Table 3:** Comparisons of spinopelvic parameters and LDI in the different groups

	Total	ASD	Non-ASD	***P***
PI (°)	54.13 ± 9.57	55.49 ± 8.22	53.26 ± 7.13	0.251
Pre LL (°)	38.71 ± 6.96	37.17 ± 7.31	38.85 ± 6.93	0.341
Pre LLL (°)	25.22 ± 4.82	23.71 ± 4.55	25.36 ± 4.83	0.177
Pre LDI	0.66 (0.60–0.71)	0.65 ± 0.11	0.66 (0.60–0.71)	0.682
Pre LDI group (low LDI, moderate LDI, high LDI), *n* (%)				0.252
Low LDI < 0.5	13 (6.5)	2 (11.8)	11 (6.0)	
0.5 ≤ moderate LDI ≤ 0.8	172 (86.0)	13 (76.4)	159 (86.9)	
High LDI > 0.8	15 (7.5)	2 (11.8)	13 (7.1)	
Pre LDI group (normal LDI, abnormal LDI ) *n* (%)				0.267
0.5 ≤ normal LDI ≤ 0.8	172 (86.0)	13 (76.5)	159 (86.9)	
Abnormal LDI (< 0.5, > 0.8)	36 (14.0)	4 (23.5)	24 (13.1)	
Post LL (°)	42.67 ± 8.51	40.35 ± 7.96	42.88 ± 8.55	0.241
Post LLL (°)	28.82 ± 5.90	27.69 ± 5.68	28.93 ± 5.93	0.410
Post LDI	0.69 ± 0.13	0.71 ± 0.19	0.68 ± 0.12	0.578
Post LDI group (low LDI, moderate LDI, high LDI), *n* (%)				0.001
Low LDI < 0.5	16 (8.0)	4 (23.5)	12 (6.6)	
0.5 ≤ moderate LDI ≤ 0.8	146 (73.0)	6 (35.3)	140 (76.5)	
High LDI > 0.8	38 (19.0)	7 (41.2)	31 (16.9)	
Post LDI group (normal LDI, abnormal LDI) *n* (%)				0.001
0.5 ≤ normal LDI ≤ 0.8	146 (73.0)	6 (35.3)	140 (76.5)	
Abnormal LDI(< 0.5, > 0.8)	54 (27.0)	11 (64.7)	43 (23.5)	

Independent-samples *t* tests, Mann-Whitney *U* test, Pearson *χ*^2^ test, Fisher’s exact test

*ASD* adjacent segment disease, LL lumbar lordosis, *LLL* lower lumbar lordosis, *LDI* lordosis distribution index

### Relationship between postoperative LDI and ASD

**Fig. 3:**
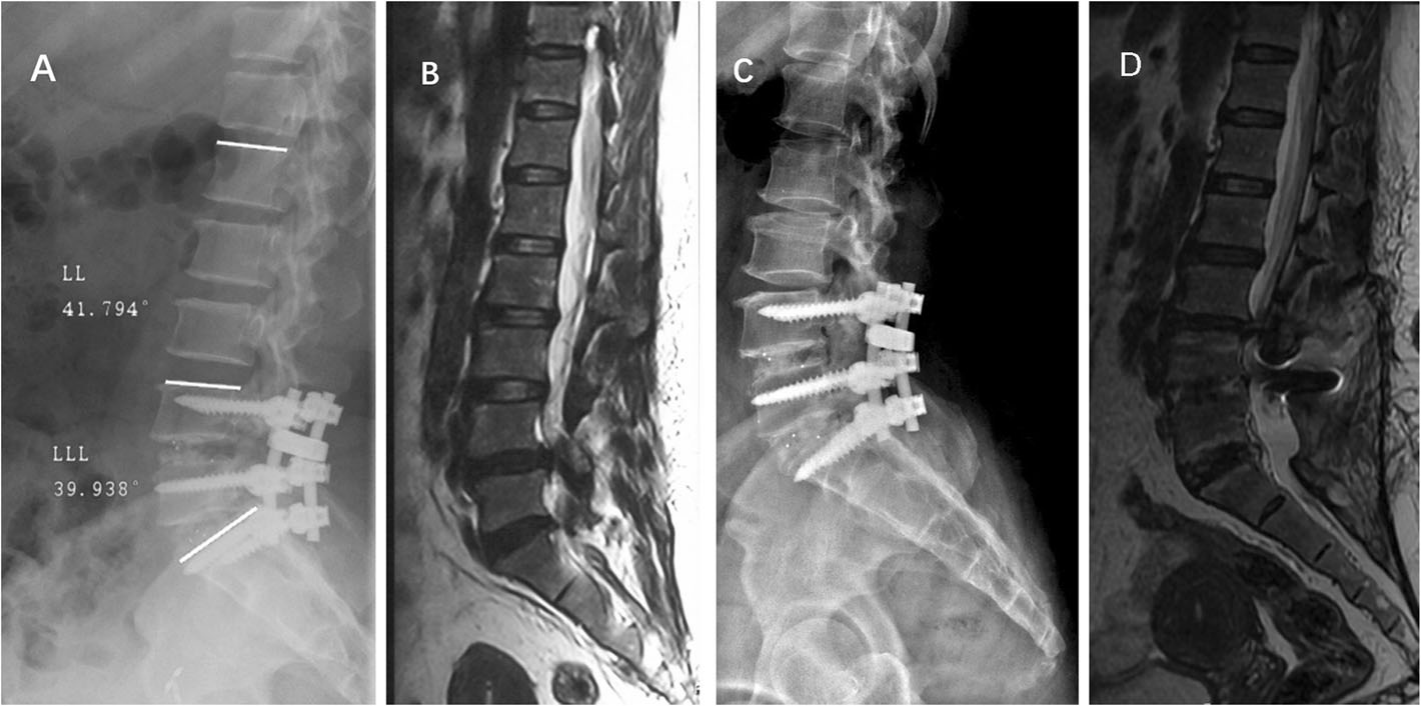
Imaging studies of ASD associated with high postoperative LDI. **a** A 47-year-old female patient underwent L4-S1 PLIF with the postoperative LL of 41.79° and LLL of 39.94°. The ultimate LDI equals to 95.57%. **b** Preoperative sagittal T2-weighted MRI scans at the L3–4 showing no or mild spinal stenosis (Imagama’s classification II) and disc degeneration of Pfirrmann classification III. **c** Postoperative neutral lateral X-ray obtained 69 months after surgery revealing retrolisthesis of L3 vertebra. **d** The final follow-up MRI scans demonstrating severe spinal stenosis (Imagama’s classification IV) and disc degeneration of Pfirrmann classification IV

With regard to the abnormal range of LDI that could be associated with the development of ASD, we attempted to integrate the low LDI subgroup and the high LDI subgroup into a single group named as abnormal LDI intergroup. The number of patients with normal LDI pre and postoperatively in the ASD group was 13 (76.5%) and 6 (35.3%), respectively. The number of patients with abnormal LDI pre and postoperatively in the ASD group was 4 (23.5%) and 11 (64.7%), respectively. Statistical analysis revealed a significant difference between normal and abnormal LDI intergroup postoperatively (*P* = 0.001), but not in the preoperative status (*P* = 0.267). A typical case is presented in Fig. 3. When the incidence of ASD among different postoperative LDI subgroups was compared, statistical significance was found not only between low and moderate LDI subgroups (25% vs 4.1%, *P* = 0.006),but also between high and moderate LDI subgroups (18.4% vs 4.1%, *P* = 0.007). However, no statistical significance was observed between low and high LDI subgroups (25% vs 18.4%, *P* = 0.859), as shown in Table 4.

**Table 4:** Comparisons of incidence of ASD among different postoperative LDI groups

	Total (***n***)	Non-ASD	ASD	***P***
Low LDI, *n* (%)	16	12 (75)	4 (25)	.006
Moderate LDI, *n* (%)	146	140 (95.9)	6 (4.1)	
Low LDI, n (%)	16	12 (75)	4 (25)	.859
High LDI, *n* (%)	38	31 (81.6)	7 (18.4)	
Moderate LDI, *n* (%)	146	140 (95.9)	6 (4.1)	.007
High LDI, *n* (%)	38	31 (81.6)	7 (18.4)	

*ASD* adjacent segment disease, *LDI* lordosis distribution index

**Fig. 4:**
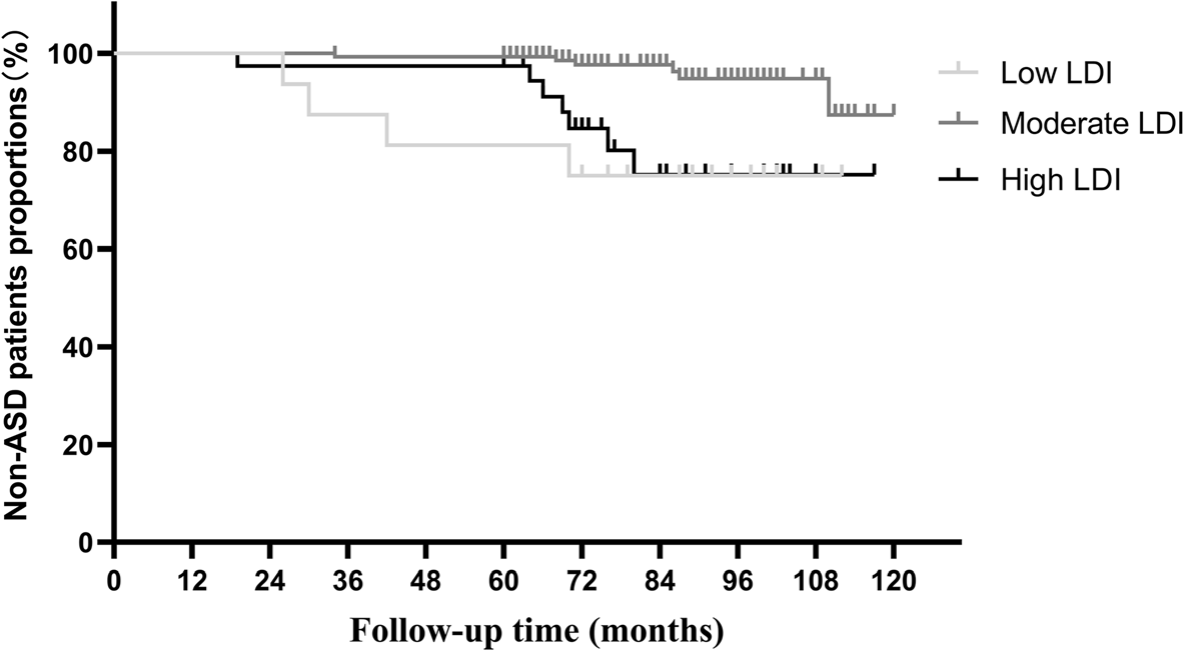
Kaplan-Meier analysis of adjacent segment disease (ASD) patients after L4-S1 posterior lumbar interbody fusion during the 120 months of follow-up period by three kinds of lordosis distribution index (LDI) groups. (log-rank test, *P* = 0.001)

Kaplan-Meier analysis survivorship by LDI subgroups was performed to assess the rate of non-ASD survival for ASD among the patients undergoing L4-S1 PLIF (Fig. 4). In low LDI subgroup, ASD-free survival was estimated to be 87.5% at 3 years and 75.0% at 6 years. In moderate LDI subgroup, ASD-free survival was estimated to be 99.3% at 3 years and 97.6% at 6 years. In high LDI subgroup, ASD-free survival was estimated to be 97.4% at 3 years and 84.5% at 6 years. The Kaplan-Meier curves exhibited a noticeable difference in ASD-free survival among the three types of LDI subgroups (log-rank test, *P* = 0.001). When the survival rates of every two kinds of LDI groups were compared, it was found out that this figure varied significantly not only between low and moderate LDI subgroups (log-rank test, *P* = 0.0012), but also between high and moderate LDI subgroups (log-rank test, *P* = 0.0005). Nevertheless, no statistical significance was observed between low and high LDI subgroups (log-rank test, *P* = 0.7223).

## Discussion

Adjacent segment disease (ASD) is a thorny sequelae after posterior lumbar interbody fusion for spinal degenerative diseases [1–8]. Many studies have reported mechanisms concerning the cause of ASD. However, the specific pathogenesis of ASD remains unclear. Liuke et al. [23] revealed the fact that BMI ≥ 25 kg/m^2^ could lead to an increased risk of lumbar disc degeneration. Bagheri et al. [24] demonstrated that the patients with higher preoperative BMI show a statistically significant increase in the risk of developing ASD. Our results were consistent with the previous studies. Normal aging and degenerative process could partially contribute to the accelerated progression of ASD. Patients’ age at the time of the index surgery has been identified as one of the most important risk factors for ASD. It has been demonstrated in multiple studies that the incidence of ASD tends to be higher based on the advancing age [21, 25]. As far as our study is concerned, however, there was no statistical relationship observed between advancing age and ASD. Masevnin et al. [26] came to a conclusion that the patients with a high PI value have a significantly higher risk of adjacent segment instability after short-segment spinal fusion. Nevertheless, there were no similar results obtained from our data. The reported incidence of ASD varies significantly, from 5.2 to 18.5% [4]. We reported a cumulative ASD incidence of 8.5%. In this series, ASD is defined as radiographic abnormalities with some new clinical symptoms that require reoperation. The incidence of ASD would be underestimated as some patients may be excluded from the ASD group.

The most important finding of this research is that the lower lumbar spinal parameter (postoperative LDI) would be a significant risk factor for the development of ASD after L4-S1 PLIF. In most studies, LL has been explored in depth among the patients with ASD. Djurasovic et al. [27] suggested that the patients developing ASD have a significantly lower level of LL. Nakashima et al. [25] drew a conclusion that obtaining appropriate LL in PLIF could play a crucial role in the prevention of ASD. Wu [28] reported that the postoperative angle of LL is 7.9° higher than the preoperative angle in patients with degenerative lumbar scoliosis after PLIF. However, there are still few studies paying attention to the construction of LDI, which tends to be affected by the ratio of LLL and LL. In our study, the angle of LL and LLL increased by 3.96°and 3.60° after surgery, respectively, while LDI increased by 0.03 postoperatively. Except for postoperative LDI subgroups, there was no significant association observed between ASD and pre LL, pre LLL, the value of pre LDI, preoperative LDI subgroups, post LL, post LLL, and the value of post LDI.

To the best of our knowledge, this is the first retrospective study conducted to investigate the potential association between postoperative LDI and ASD after L4-S1 PLIF, in which LLL was constant for the instrumentation of L4-S1. In a study of 222 patients with degenerative spinal scoliosis, Yilgor et al. [16] developed a new approach to GAP score, where LDI is treated as a critical component, so as to analyze the spinopelvic alignment and predict the mechanical complications following adult spinal deformity surgery. In addition, it has been reported that the integration of relative lumbar lordosis (RLL) and lordosis distribution index (LDI), compared with PI-LL, may be contributory to reducing the rates of mechanical complications and improving long-term HRQOL [29]. Ohba et al. [30] came to the conclusion that postoperative LDI plays a significant role in the prevention of excess reciprocal progression of TK, which could lead to proximal junctional kyphosis. These studies serve as a reminder for all spine surgeons of the role played by LDI in spinal biomechanics. In this paper, we clarified the relationship between postoperative LDI and ASD after L4-S1 PLIF. The patients with abnormal postoperative LDI (LDI < 0.5, LDI > 0.8) were found to be more likely to develop ASD compared to those with normal postoperative LDI (0.5 ≤ LDI ≤ 0.8). The incidence of ASD in low LDI group and high LDI group was 25% and 18.4% respectively, significantly higher than that (4.1%) in moderate LDI group. It is speculated that the abnormal LDI could cause changes to the physiological distribution of loads and lead to an increase in biomechanical stress at the level adjacent to the fused segment, thus accelerating disc degeneration and intervertebral instability [31].

Though the patients with abnormal LDI exhibited a lower rate of ASD-free survival than the patients with normal LDI, low LDI appears to be more concerning than high LDI as revealed by the Kaplan-Meier survivorship analysis. Menezes-Reis et al. [32] reported that the hypolordosis of lumbar spine was associated with a higher frequency of ASD. It has been discovered that postoperative loss of lumbar lordosis could cause excessive mobility and increase biomechanical stress [33]. This in turn causes premature degeneration of the facet joints. As the facet degenerates, the translation of the adjacent segment may occur to produce listhesis. This is consistent with our finding that the patients with low LDI were more susceptible to the development of ASD.

The design of our study had several potential limitations. Firstly, it is a retrospective study with inherent difficulties encountered for studying ASD. Further prospective studies are deemed necessary to validate the relationship between ASD and postoperative LDI. Secondly, this is a single-center study; hence, the results may not be representative of all patients undergoing L4-S1 PLIF. Thirdly, the patients were positioned on the lateral decubitus position when taking a radiograph. However, the relationship between postoperative LDI and ASD needs to be further investigated in the standing position.

## Conclusions

In this study, an investigation was conducted into the relationship between postoperative LDI and ASD in a total of 200 patients treated with L4-S1 PLIF at an average follow-up of 84 months. BMI is a risk factor for ASD in the patients undergoing L4-S1 PLIF for degenerative spine diseases. Statistically the patients with an abnormal postoperative LDI were more likely to develop ASD than those with normal postoperative LDI. Moreover, the patients with low postoperative LDI were at greater risk of developing ASD than those with high postoperative LDI over time. Meanwhile, it was suspected that obtaining appropriate postoperative LDI in L4-S1 PLIF could play a crucial role in the prevention of ASD.

## Data Availability

The patients’ data were collected in the Chinese PLA General Hospital. The datasets used and/or analyzed during the current study are available from the corresponding author on reasonable request.

## Abbreviations

LDI: Lordosis distribution index
PLIF: Posterior lumbar interbody fusion
ASD: Adjacent segment disease
LL: Lumbar lordosis
LLL: Lower lumbar lordosis
BMI: Body mass index
